# Virtual Reality Reminiscence Therapy in Dementia Care: Scoping Review of Research

**DOI:** 10.2196/73539

**Published:** 2025-08-06

**Authors:** Maria Matsangidou

**Affiliations:** 1 CYENS Centre of Excellence, Lellou Demetriades, Plateia Dimarchou 1, Nicosia, 1016, Cyprus, 357 22 747575

**Keywords:** dementia, virtual reality, reminiscence therapy, nonpharmacological interventions, cognitive stimulation, emotional stimulation

## Abstract

**Background:**

Dementia is a progressive neurological disorder affecting cognitive and social functioning, posing challenges for patients and caregivers. Traditional medications often have adverse effects, emphasizing the need for nonpharmacological options such as reminiscence therapy (RT). Virtual reality (VR) has emerged as a promising tool in dementia care, providing immersive experiences that stimulate memory, enhance emotional well-being, and reduce the behavioral and psychological symptoms of dementia.

**Objective:**

This scoping review assesses the feasibility and implementation challenges of delivering RT via VR in dementia care. Specifically, it examines the types of VR systems used, their therapeutic benefits, and the barriers to their adoption.

**Methods:**

We screened 5 electronic libraries: Google Scholar, ACM Digital Library, IEEE Xplore, MEDLINE, and PubMed. Studies published between 2000 and 2025 were included if they examined the use of VR for RT in people with dementia. Data were charted based on PRISMA-ScR (Preferred Reporting Items for Systematic Reviews and Meta-Analyses Extension for Scoping Reviews) guidelines and analyzed thematically for feasibility, VR system type, therapeutic effects, and implementation considerations.

**Results:**

A total of 15 studies met the inclusion criteria. The findings indicate that VR is feasible and well-accepted among people with dementia, fostering high engagement with minimal adverse effects. Fully immersive VR systems, which use head-mounted displays, are the most frequently used, while semi-immersive alternatives with large screens provide a more cost-effective option. RT via VR has been shown to improve reminiscence, enhance mood, and encourage social interaction. However, its impact on cognitive function remains inconclusive. Significant barriers to implementation include high costs, limited availability of VR infrastructure in care, and the need for specialized caregiver training.

**Conclusions:**

RT via VR presents a promising advancement in dementia care. Future research should focus on developing cost-effective, scalable VR solutions, designing personalized VR experiences tailored to individual needs, and creating structured training programs for caregivers. Longitudinal studies are necessary to determine the long-term therapeutic effects of VR compared to traditional RT.

## Introduction

### Background

Dementia is a progressive neurological disorder and an umbrella term for conditions that lead to the deterioration of cognitive and social functioning. It impacts various mental faculties, including memory, problem-solving, orientation, comprehension, calculation, learning capacity, language, and judgment [1]. People with dementia often experience behavioral and psychological symptoms of dementia (BPSD), including mood disturbances, aggression, irritability, apathy, and emotional dysregulation, which further reduce their quality of life and place significant burdens on caregivers [2,3].

Worldwide, dementia affects a rapidly growing population. Only in 2021, the World Health Organization estimated that over 55 million people were living with dementia, a number expected to rise to 82 million by 2030 and 139 million by 2050 [1]. It is currently the seventh leading cause of death worldwide and a major cause of disability and dependency among older adults [1]. This rising prevalence underscores the urgent need for interventions, as dementia imposes significant physical, psychological, social, and economic burdens [2].

While no cure exists, treatments aim to alleviate symptoms and improve the quality of life for people with dementia. Pharmacological treatments, such as neuroleptic or sedating medications, have historically been overused despite their association with adverse effects, including accelerated cognitive decline, cardiovascular issues, infections, and emotional distress [4,5]. Consequently, nonpharmacological interventions have gained prominence as preferred approaches for addressing dementia symptoms. Among these, reminiscence therapy (RT) has emerged as a particularly effective psychosocial intervention. RT involves structured recall and discussion of past experiences using prompts such as photographs, music, personal objects, and storytelling [6]. Evidence suggests that RT can improve mood, reduce agitation, and enhance social interaction in people with dementia [6,7].

The application of RT varies significantly across studies, with interventions delivered in both personalized and nonpersonalized formats. Some studies combined both approaches [8], while others focused exclusively on either personalized RT [9,10] or nonpersonalized RT [6,11]. RT methods, such as group storytelling, memory books, multisensory activities, and music therapy, have been widely used [9,12]. Meanwhile, digital RT, which integrates digital apps, video games, and web-based platforms, has introduced novel ways of engaging people with dementia [8,10,11]. Such digital interventions have shown promise in increasing engagement and reducing social isolation [12]. However, challenges such as cost, feasibility, and inconsistent therapeutic outcomes remain [6].

### Emerging Role of Virtual Reality in RT

Recent technological advancements, particularly virtual reality (VR), have expanded the possibilities for nonpharmacological dementia care [13,14]. VR creates immersive, computer-generated environments that simulate real-world experiences, engaging users’ senses through visual, auditory, tactile, and even olfactory stimuli [15]. These environments enable people with dementia to transcend physical limitations and explore familiar or calming settings, such as natural landscapes or culturally significant landmarks [14,16,17]. By simulating personalized environments, VR interventions have demonstrated the potential to reduce anxiety, depression, aggression, and social withdrawal while promoting cognitive engagement and emotional well-being [13,14,18-20].

Studies show that personalized VR experiences, tailored to an individual’s preferences and abilities, have been linked to improved cognitive functions, such as memory recall and spatial navigation, as well as reductions in agitation and emotional dysregulation [21]. Moreover, VR-based interventions offer scalable, customizable solutions that can be adapted to the diverse needs of people with dementia while minimizing mobility challenges [14,18,22-25].

Despite these promising findings, questions remain regarding the feasibility and the limitations of integrating VR into RT for dementia care. While several systematic and scoping reviews have examined the use of virtual reality reminiscence therapy (VRRT) for older adults or people with cognitive impairments [26,27], none, to the best of our knowledge, have focused exclusively on people with dementia. Moreover, existing reviews often conflate diverse populations, such as cognitively healthy older adults, those with mild cognitive impairment, and individuals with dementia, limiting the specificity and relevance of their conclusions to people with dementia. Further on the above, these prior works also tend to overlook important dimensions such as the comparative use of semi-immersive virtual reality (SI-VR) versus fully immersive virtual reality (FI-VR) systems, the role of content personalization, and implementation barriers in real-world care. This review addresses these critical gaps by focusing solely on people with dementia and providing a comprehensive synthesis of empirical and experimental studies that evaluate both the therapeutic impact and practical challenges of VR-based RT. As VR technology continues to evolve, further research is needed to determine its optimal application, long-term effects, and best practices for integration in dementia care. This study examines the implementation of VR in RT for people with dementia residing in long-term care facilities, hospital environments, or community settings over the past 2 and a half decades (2000‐2025). By synthesizing findings from empirical and experimental research, the review addresses critical research questions (RQs), including the following:

- RQ1. How feasible is integrating VR into RT for dementia care?
- RQ2. What are the outcomes of VRRT?
- RQ3. What limitations currently hinder the application of VR in RT?
- RQ4. What are the potential future directions for research and implementation of VR in RT?

## Methods

### Literature Review Strategy

The electronic databases Google Scholar, ACM Digital Library, IEEE Xplore, MEDLINE, and PubMed were searched in January 2025 using a combination of search terms designed to capture studies on the use of VR in RT for people with dementia. Three core concept clusters were applied: term A included “dementia,” and term B included “reminiscence” OR “reminiscence therapy,” and term C included “virtual reality.” A filter was applied to include only studies that were published between 2000 to 2025. The reference lists of articles that met the eligibility criteria were further perused to identify additional studies that may fall within the scope of this review.

### Inclusion and Exclusion Criteria

Studies eligible to be included in this review had to meet the following inclusion criteria: (1) human participants were involved, (2) the full article was written in English, and (3) papers studied VR used for RT in dementia. The exclusion criteria were (1) publications where the study of VR used for RT in dementia was not the primary aim of the study, (2) publications that were not original studies (ie, review articles, letters, medical hypotheses, etc), (3) publications that presented trials studying subjects with no dementia, (4) duplicate publications, (5) publications whose abstract was not accessible, and (6) publications whose full text could not be obtained.

### Data Collection Process

Following the identification of eligible publications, all relevant data were collected using a structured coding scheme in an Excel (Microsoft Corp) file. The data collected included titles, sample size, type of dementia, instruments used, methodology, and findings. Additional fields specific to studies involving VR included the type of VR system (nonimmersive, semi-immersive, or fully immersive), VR content or intervention, feasibility, limitations, and future directions.

### Data Synthesis and Analysis

This study used aggregated data where possible, per the PRISMA-ScR (Preferred Reporting Items for Systematic Reviews and Meta-Analyses Extension for Scoping Reviews) guidelines (Checklist 1: PRISMA-ScR). Key domains extracted included feasibility, therapeutic outcomes (emotional, cognitive, and social), and implementation barriers, allowing for cross-study comparison and pattern identification.

## Results

### Search Results

**Figure 1.**
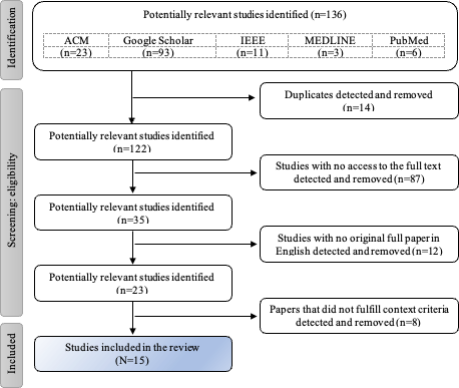
Flowchart detailing the process used to identify and select the papers included in the analysis.

This search strategy yielded the identification of 136 articles. Following the eligibility assessment, 121 articles were excluded. In total, 15 papers satisfied the inclusion criteria and were used for this review (Table 1). Figure 1 illustrates the study selection process.

**Table 1.** Sample characteristics, dementia types, and severity levels.

	Study	Sample	Type of dementia	Level of dementia	Experimental design	RT^a^ intervention
1	Appel et al, 2020 [16]	8 females/2 males, mean age: 86.5 years	AD^b^, FD^c^, MD^d^, and VD^e^	Mild to severe	Mixed methods design, and combined quantitative and qualitative data	A single up to 20-minute FI-VR^f^ RT session, focusing on managing BPSD^g^
2	Appel et al, 2024 [28]	45 females/24 males, age: 65+ years	N/A^h^	Mild to severe	Mixed methods design, randomized controlled trial, and combined quantitative and qualitative data	1‐3 FI-VR sessions lasting up to 20 minutes each, focusing on managing BPSD
3	Brimelow et al, 2020 [29]	9 females/4 males, mean age: 66‐93 years	N/A	Mild to moderate	Mixed methods design; combined quantitative observation and qualitative interviews post a single VR^i^ session	A single up to 5-minute FI-VR RT individual or group session, focusing on apathy and mood
4	Coelho et al, 2020 [30]	6 females/3 males, mean age: 85.6 years	N/A	N/A	Mixed methods design, and combined quantitative and qualitative pre- and postdata	A total of 4 sessions lasting up to 15 minutes each of personalized FI-VR tailored to participants’ psychological needs
5	Ferguson et al, 2020 [31]	22 females/3 males, mean age: 85 years	AD, MD, and VD	N/A	Mixed methods design, and combined quantitative and qualitative data	A single up to 30-minute FI-VR RT session, focusing on the feasibility of the system
6	Huang and Yang, 2022 [32]	11 females/9 males, mean age: 79 years	N/A	Mild to moderate	Longitudinal observational study design, and quantitative data	A 10 to 12-minute FI-VR RT session held twice a week for 3 months, targeting cognitive function, global status, and depression
7	Kim et al, 2021 [33]	10 females, mean age: 85.80 years	N/A	Mild	Mixed methods design, and combined quantitative and qualitative data, including a survey to gather information about the psychological needs of each patient, to customize the system	1‐2 sessions lasting 20‐30 minutes each of personalized FI-VR tailored to participants’ psychological needs
8	Klein et al, 2018 [34]	3 females/3 males, mean age: 74.67 years	N/A	N/A	Qualitative methods design, and combined observational data and multiple focus groups before the experiment to gather information about the needs of people with dementia, to design the system	A single 12‐20 minute FI-VR RT session, focusing on efficacy and acceptance of the system
9	Manera et al, 2016 [35]	12 females/17 males, mean age: 76.3 years	AD and MD	N/A	Within-subject design, and combined quantitative and task performance data	A single 10-minute SI-VR^j^ and controlled session, focusing on security, comfort, apathy, and anxiety
10	Moyle et al, 2018 [36]	7 females/3 males, mean age: 89 years	AD	N/A	Mixed methods design, and combined quantitative and qualitative data	A single up to 15-minute FI-VR RT session, focusing on engagement, apathy, and mood states
11	Ng et al, 2023 [17]	26 females/11 males, mean age: 65+ years	N/A	Mild	Nonrandomized controlled trial methods design and quantitative data	A single up to 12-minute FI-VR RT session, focusing on systems evaluation
12	Rose et al, 2021 [37]	2 females/6 males, mean age: 69.63 years	AD, FD, HD, and MD	Mild to severe	Mixed methods design, and quantitative observations and qualitative pre- and postdata	2 FI-VR RT sessions, lasting up to 15 minutes each, which focus on natural environments, aiming to reduce BPSD
13	Saredakis et al, 2020 [38]	10 females/7 males, mean age: 87.3 years	N/A	Mild to moderate	Mixed methods design and quantitative data	A single up to 20-minute FI-VR RT session, focusing on apathy
14	Saredakis et al, 2021 [39]	28 females/15 males, mean age: 84.8 years	N/A	N/A	Nonrandomized controlled trial methods design, and combined quantitative and objective data	A total of 3 FI-VR RT sessions, lasting up to 20 minutes each, focusing on apathy, cognition, depression, and quality of life
15	Tabbaa et al, 2019 [40]	2 females/6 males, mean age: 69.63 years	AD, MD, and VD	Mild to severe	Mixed methods design, and combined quantitative observations and qualitative pre- and postdata. A focus group was conducted before the experiment to gather information about the needs of people with dementia, to design the system	A total of 2 FI-VR RT sessions, lasting up to 15 minutes each, focusing on the technical aspects of the system, as well as its feasibility, acceptability, and practicality

a RT: reminiscence therapy.

b AD: Alzheimer disease.

c FD: frontotemporal dementia.

d MD: mixed dementia.

e VD: vascular dementia.

f FI-VR: fully immersive virtual reality.

g BPSD: behavioral and psychological symptoms of dementia.

h N/A: not available.

i VR: virtual reality.

j SI-VR: semi-immersive virtual reality.

k HD: Huntington disease.

### Study Characteristics

The present review analyzes 15 studies, and the participant demographics include both male and female people with dementia, with mean ages ranging from 65 to 89 years. Several studies focus on specific types of dementia, including Alzheimer disease, vascular dementia, frontotemporal dementia, and mixed dementia, whereas others do not specify the type of dementia examined. The studies reflect a range of dementia severity from mild to severe, though the majority emphasize mild to moderate cases [17,27,38]. A summary of the demographics of the reviewed studies is presented in Table 1.

### Types of VR Systems, Levels of Immersion, and Virtual Environments Used in Dementia RT

The reviewed studies implemented 2 levels of VR immersion, as detailed in Table 2, with FI-VR being the most commonly used method, present in 12 of 16 studies. FI-VR necessitated the use of head-mounted displays (HMDs), such as Samsung Gear VR, Oculus Rift, HTC VIVE, Oculus Quest, Oculus Go, and Windows Mixed Reality headsets, to provide 360-degree panoramic views and interactive experiences [27,33,39]. For instance, one study used the Samsung HMD Odyssey Windows Mixed Reality with Leap Motion sensors (LM-010), allowing people with dementia to interact with the VR environment using hand gestures, including controlling seasonal transitions and engaging with animated objects [33]. Similarly, 2 other studies used the HTC VIVE Pro to simulate a familiar home environment, incorporating daily life objects and home appliances [17,32]. Several studies used Samsung Gear VR with a smartphone, allowing people with dementia to immerse themselves in calming virtual environments, including underwater scenes, travel destinations, and snowy landscapes [27,37,40].

In 2 reviewed studies, the content was mirrored on an external flat screen, allowing caregiver participation in providing reassurance and guidance during VR sessions [37,40]. Additionally, 1 study created their custom HMD featuring 180-degree projection to simulate time travel through various historical periods, including Berlin and Paris in the 20th century [34].

SI-VR was used in 3 studies where people with dementia experienced virtual environments on large projection screens rather than HMDs [35,36]. This approach allowed for limited interaction through a mouse, touchscreen, or sensor-based technology, while still providing an engaging experience. For example, 1 study used stereoscopic 3D screens paired with Volfoni Edge 1.2 active 3D LCD shutter glasses to deliver an SI-VR experience [35]. Lastly, 2 additional studies incorporated SI-VR, projecting the virtual forest onto a large display screen, with sensor-based interactions that allowed people with dementia to explore the river, trees, and surrounding environment [36] as well as familiar and unfamiliar environments [30,33]. Nonimmersive VR was not featured in any of the studies examined.

**Table 2.** Apparatus and virtual environments.

	Study	Type of VR^a^	Virtual environment	Equipment
1	Appel et al, 2020 [16]	FI-VR^b^	Rocky lakeshore, forests, floating icebergs, and beaches	Samsung Gear VR and Sennheiser HD^c^ 221 headphones
2	Appel et al, 2024 [28]	FI-VR	Calming in distinctive ways and nature visualization	Oculus Go with built-in and external Sennheiser HD 221 headphones
3	Brimelow et al, 2020 [29]	FI-VR	Underwater themes, beaches, farmyard animals, travel destinations, and snowscapes	Samsung Galaxy S7 and Samsung Gear VR headset
4	Coelho et al, 2020 [30]	FI-VR	Forests, beaches, cathedrals, childhood homes, workplaces, and religious venues	Samsung Gear VR and Oculus Rift VR
5	Ferguson et al, 2020 [31]	FI-VR	Beach scene	Mirage Solo with Daydream Business Edition
6	Huang and Yang, 2022 [32]	FI-VR	1960‐1980 Taiwan: historical residence, radio, photo album, and feeding chickens	VIVE Pro
7	Kim et al, 2021 [33]	FI-VR	Streets of Memory, Nostalgic Youth, Homely Hometown, and Where I Want to Go	Leap Motion sensors and Samsung Odyssey Windows Mixed Reality Headset
8	Klein et al, 2018 [34]	Between SI-VR^d^ and FI-VR	Time travel: Berlin (1970‐1949), movie stars (1950s-1960s), television shows, and Paris in the 20th century	Custom-built HMD^e^ (180-degree projection)
9	Manera et al, 2016 [35]	SI-VR	People	Barco OverView OLSF-721^f^ full HD 3D stereoscopic LED video wall, Volfoni Edge 1.2 active 3D LCD shutter glasses
10	Moyle et al, 2018 [36]	SI-VR	Forest	Large screen
11	Ng et al, 2023 [17]	FI-VR	Early home environment: appliances and daily necessities	HTC Vive Pro and Leap Motion sensor
12	Rose et al, 2021 [37]	FI-VR	Nature and urban, forest, countryside, sandy or rocky beaches, and a cathedral	Samsung Gear VR and Samsung Galaxy S6
13	Saredakis et al, 2020 [38]	FI-VR	Personalized VR videos and places via Google Street View	Oculus Go
14	Saredakis et al, 2021 [39]	FI-VR	Personalized VR videos and places via Google Street View	Oculus Quest
15	Tabbaa et al, 2019 [40]	FI-VR	Cathedral, forest, sandy beach, rocky beach, and countryside	Samsung Gear VR and Samsung Galaxy S6 mobile phone

a VR: virtual reality.

b FI-VR: fully immersive virtual reality.

c HD: high definition.

d SI-VR: semi-immersive virtual reality.

e HMD: head-mounted display.

f OLSF: OverView LED Slim Front access.

### VRRT and the Role of Nature, Nostalgic Memories, and Social Themes

Overall, our review findings indicate that the virtual environments designed for RT were mainly created to be calming and engaging, with a strong emphasis on nature, nostalgic memories, and social themes (Table 2). RT via VR aims to create immersive, emotionally meaningful experiences that promote relaxation and memory recall and enhance engagement for people with dementia. Numerous studies demonstrated that virtual environments featuring peaceful natural landscapes, historical experiences, and familiar settings contribute significantly to the emotional and psychological well-being of people with dementia [16,17,27,31,32,34,36,38].

One of the most widely used themes in RT via VR was nature, as research indicates that natural landscapes help induce relaxation and reduce agitation in people with dementia [16,17,27,36,37]. It was found that people with dementia who experienced virtual forests, lakes, and beaches reported reduced anxiety and enhanced emotional engagement [16]. It was further highlighted that participants displayed lower distress levels when immersed in nature-based VR experiences, particularly those incorporating sensory elements such as bird sounds, flowing water, and open landscapes [28,37].

Nostalgic memories were another central element in RT in VR, as many virtual environments replicate meaningful locations linked to the people with dementia’s past that can trigger autobiographical memories [27,31,32,34,38]. People with dementia responded positively to virtual environments that resembled places from their younger years, leading to enhanced memory recall and more profound personal engagement with caregivers and peers. These places included simulated childhood homes, nostalgic town streets, familiar workplaces, and meaningful travel destinations, including time travel experiences [27,30,33,37,40]. For example, people with dementia were immersed in “Streets of Memory,” a VR representation of old neighborhoods and markets [33], past workplaces such as farms and factories [30], cathedrals and holy places [40], natural landscapes such as beaches and forests that evoked memories of youth [27,31,37,40], and rural settings from 1950‐1970 [32,34].

Finally, it was observed that people with dementia were more likely to participate in discussions and share personal experiences when the virtual environments replicated familiar social places [30,31,33,37,40]. These places included, for instance, a VR Christmas dinner [31], a cathedral church service [37], a retired teacher who experienced a VR classroom [30], a familiar-looking café [33], and a personalized Google street view experience, where people with dementia could visit places they remember, such as old neighborhoods, vacation spots, and religious sites [38,39].

### Feasibility and Impact of VRRT

Overall, the reviewed studies consistently demonstrated that VR for RT is feasible and can have a positive impact on enhancing emotional well-being and social engagement in people with dementia (Table 3). Several studies confirmed that people with dementia could successfully engage with VR without experiencing significant adverse effects [27,28,30,33,40]. However, minor side effects, including dizziness, nausea, and discomfort, were reported [27,33,38], though these were temporary and did not affect the overall feasibility.

Further to the above, several studies reported that people with dementia were able to engage with VR environments, often requiring only initial guidance or passive supervision. While explicit data on caregiver workload was limited, these findings suggest the potential for VRRT to be implemented with manageable facilitation demands in structured care settings [27,28,30,40].

Moreover, the flexibility of VR delivery formats, specifically the availability of both fully immersive and semi-immersive systems, emerged as a significant facilitator of feasibility. Fully immersive systems, such as HMDs, provide a deeper sense of presence and sensory engagement, which can enhance therapeutic outcomes. However, these systems often come with higher costs and setup requirements. In contrast, semi-immersive systems using large screens or projection displays offer a more accessible and logistically manageable alternative, particularly beneficial for individuals with mobility limitations or in resource-constrained care settings. This dual-modality approach increases the adaptability of VRRT, making it feasible across a range of environments, from long-term residential facilities to community-based programs [27,30,38,40]. In summary, the findings from multiple studies indicate that VRRT is feasible and well-tolerated, with adaptable delivery formats that are suitable for various care settings.

**Table 3.** Reported side effects, feasibility, and outcomes of reminiscence therapy via virtual reality.

	Study	Instruments	Reported side effects	Feasibility or tolerance	Results
1	Appel et al, 2020 [16]	NPI^a^, Confusion Assessment Method score, Montreal Cognitive Assessment, MMSE^b^, recording instances of BPSD^c^, and semistructured interviews.	Temporary feelings of dizziness and nausea.	People with dementia tolerated VR^d^ well. VR is a feasible nonpharmacological intervention in acute care hospitals.	VR is a deployable, scalable, nonpharmacological solution for managing BPSD, which can significantly help dementia patients and their caregivers.
2	Appel et al, 2024 [28]	Quality of Life in Late-Stage Dementia Scale, nurses’ daily notes for BPSDs and falls, and structured observations and interviews.	Two people with dementia experienced nervousness, anxiety, confusion, or disorientation, and 1 person with dementia experienced nausea.	People with dementia tolerated VR well. VR is a feasible nonpharmacological intervention in acute care hospitals.	VR is a safe, well-tolerated, and enjoyable nonpharmacological solution that can facilitate RT and significantly reduce aggressiveness in people with dementia.
3	Brimelow et al, 2020 [29]	PEAR^e^, OERS^f^, and structured observations and interviews.	Two people with dementia and impaired vision reported symptoms of cybersickness. One person with dementia found the headset slightly uncomfortable. Another person with dementia reported feeling “giddy,” which was temporary upon device removal.	Mobile-based VR is feasible. People with dementia found VR enjoyable with low levels of physical and emotional discomfort.	The study found no impact on OERS measures; no significant increase in fear or anxiety. Reminiscence was observed in 6 of the 9 verbally communicative residents.
4	Coelho et al, 2020 [30]	Disability in daily activities (Barthel Index and Lawton and Brody Scale), Montreal Cognitive Assessment, Global Deterioration Scale, Cornell Scale for Depression in Dementia, NPI, SSQ^g^, and EUROHIS-QOL-8.	N/A^h^	A feasible solution with no significant adverse effects related to simulator sickness or psychological and behavioral symptoms.	RT^i^ via VR can benefit people with dementia, who are actively engaged in the sessions and share memories. No significant psychological or behavioral symptom changes were found.
5	Ferguson et al, 2020 [31]	Functional Assessment Staging Scale, PAINAD^j^, and semistructured interviews.	Two people with dementia experienced worsened BPSD after VR exposure. Their PAINAD scores increased, indicating discomfort, distress, or pain.	VR is safe and enjoyable.	VR provides meaningful activity and enhances the quality of life for people with dementia.
6	Huang and Yang, 2022 [32]	Cognitive Abilities Screening Instrument, MMSE, Global status by Clinical Dementia Rating, and Depressive symptoms by the Center for Epidemiological Studies of Depression.	N/A	Feasible and well-tolerated.	RT via VR can improve mood and help preserve cognitive function in people with dementia during the intervention period.
7	Kim et al, 2021 [33]	MMSE, Activities of Daily Life, VR immersion scale, and observations.	Two people with dementia reported dizziness or nausea during VR exposure.	VR therapy was feasible and provided high satisfaction and immersion.	VR can be used to treat BPSD.
8	Klein et al, 2018 [34]	Semistructured interviews and observations.	N/A	Feasible when special consideration is given to choosing personally relevant and engaging content, as well as the therapy’s contextual factors.	VR can enrich traditional RT, foster conversations, and support positive interactions between caregivers and people with dementia.
9	Manera et al, 2016 [35]	MMSE, Clinical Dementia Rating Scale-Sum of Box scores, diagnostic criteria for apathy in clinical practice, and attention tasks.	Low levels of discomfort, anxiety, and fatigue.	People with dementia reported high satisfaction and security.	VR can have a positive impact on people with dementia experiencing apathy.
10	Moyle et al, 2018 [36]	OERS, PEAR, semistructured interviews, and structured observations.	Some participants experienced mild fear or anxiety during VR.	Feasible and well-tolerated.	VR was perceived to have a positive effect on people with dementia. However, compared to the normative sample, a greater level of fear or anxiety was observed during VR. It may have the potential to improve quality of life.
11	Ng et al, 2023 [17]	N/A	Dizziness as an effect of VR teleporting.	Feasible but complex.	The study supports the use of VR for RT in people with dementia.
12	Rose et al, 2021 [37]	OERS, OAS-MNR^k^, St Andrews Sexual Behavior Assessment, time exposed, and semistructured interviews.	One person with dementia reported dizziness due to the frequent movement of the headset to and from their eyes.	The study provides evidence of the clinical feasibility of VR implementation in health care settings.	VR can enhance the emotional well-being of people with dementia.
13	Saredakis et al, 2020 [38]	Psychogeriatric Assessment Scale, AES^l^, SSQ, Slater-Usoh-Steed Presence Questionnaire, Phonemic and Semantic Verbal Fluency Tasks, expectations or enjoyment measure, and structured interview.	A total of 35% (6/17) of participants experienced temporary side effects such as discomfort around the cheekbone, nausea, and dizziness.	RT via VR is highly feasible.	People with dementia showed improved semantic scores immediately after using VR for RT. Those with higher levels of apathy demonstrated the greatest cognitive improvements after VRRT^m^.
14	Saredakis et al, 2021 [39]	AES, Addenbrooke Cognitive Examination III, Geriatric Depression Scale, Quality of Life in Alzheimer Disease, Three-Item Loneliness Scale, SSQ, and structured observations and interviews.	Two people with dementia reported after-effects (headache and head feeling heavy) that occurred in the evening following a morning VR session.	VR can be implemented in an aged care setting with appropriate protocols in place.	People with dementia enjoyed RT via VR.
15	Tabbaa et al, 2019 [40]	OERS, OAS-MNR, semistructured interviews, and observational notes.	N/A	Feasible and well-tolerated.	VR enhanced the emotional well-being of people with dementia, with effects lasting for a short time after the session. VR also facilitated emotional openness between caregivers and people with dementia.

a NPI: Neuropsychiatric Inventory.

b MMSE: Mini-Mental State Exam.

c BPSD: behavioral and psychological symptoms of dementia.

d VR: virtual reality.

e PEAR: Person-Environment Apathy Rating.

f OERS: Observed Emotion Rating Scale.

g SSQ: Simulator Sickness Questionnaire.

h N/A: not available.

i RT: reminiscence therapy.

j PAINAD: Pain Assessment in Advanced Dementia.

k OAS-MNR: Overt Aggression Scale-Modified for Neurorehabilitation.

l AES: Apathy Evaluation Scale.

m VRRT: virtual reality reminiscence therapy.

### VRRT and Its Impact on Emotional Well-Being

Emotional well-being is a vital aspect of dementia care, as people with dementia often face mood disturbances, anxiety, depression, and apathy. These issues are collectively known as the BPSD and can severely affect the quality of life of people with dementia [14,38]. Our review indicates that RT delivered through VR has been shown to have a positive impact on the BPSD, with numerous studies highlighting these benefits. Specifically, studies reported that the use of VR improved mood, reduced agitation, and increased engagement among people with dementia [27,31,35-38]. Additionally, RT via VR has been shown to alleviate emotional distress and psychological discomfort for both people with dementia and their caregivers, who were found to experience significant emotional relief and relaxation during VR reminiscence sessions [31,40], suggesting that VR can serve as a stress-reducing intervention.

One of the primary reasons RT via VR can enhance emotional well-being is its ability to immerse people with dementia in calming, familiar, or personally meaningful environments. Specifically, studies found that people with dementia who experienced VR environments depicting natural landscapes, such as forests, lakes, and beaches, exhibited increased relaxation and reduced anxiety [16,17,36]. Similarly, people with dementia who engaged in VR sessions featuring peaceful, scenic locations such as underwater themes, farmyards, or travel destinations displayed fewer signs of agitation and distress [27,34,38].

Furthermore, as with traditional RT, RT via VR can evoke deeply personal and emotionally meaningful experiences, which contribute to a sense of identity, self-awareness, and emotional fulfillment. It was observed that during RT in VR, people with dementia were able to restore memories from their past, triggering emotions tied to nostalgia, love, and belonging [39,40]. This emotional re-engagement often strengthens feelings of self-worth and dignity, which are crucial for maintaining well-being in dementia care [27]. To further support the above, another study found that VR reminiscence experiences elicited high levels of emotional satisfaction and security, with participants displaying more expressions of happiness and comfort compared to non-VR RT [35,37].

### VRRT and Its Impact on Social Engagement

Social engagement is a crucial aspect of well-being for people with dementia, as it helps reduce loneliness, improve emotional stability, and strengthen relationships with caregivers and family members [38,41]. RT via VR has been shown to facilitate meaningful interactions by encouraging people with dementia to share personal experiences, engage in conversations, and participate in immersive social settings. Studies have consistently highlighted the ability of RT via VR to stimulate both verbal and nonverbal interactions, resulting in enhanced social connections [31,35,36,40]. To further support this, multiple studies observed that people with dementia who participated in VR reminiscence sessions were more likely to express emotions, initiate conversations, and reflect on past experiences. This enabled caregivers to gain deeper insights into their personal stories [39,40]. Additionally, research indicated that RT via VR enabled group discussions and storytelling, which encouraged people with dementia to comment on each other’s experiences and engage in collective reminiscing, which reinforced social bonds [28,34,35].

Beyond verbal interactions, RT through VR also boosts emotional engagement, which plays a crucial role in maintaining social relationships [42]. Our review found that people with dementia who participated in RT via VR showed increased smiling, laughter, and eye contact, indicating greater emotional connectivity with those around them [36,39]. These findings imply that VRRT not only triggers memories but also fosters present-moment social interactions that contribute to emotional well-being.

Another significant way RT via VR enhances social engagement is by reducing social withdrawal and apathy, common symptoms in dementia that often lead to isolation [38]. A study found that people with dementia who participated in RT via VR exhibited lower levels of social withdrawal and depression, indicating that immersive experiences can encourage active participation in social interactions [27,37]. This aligns with findings from another study [40], which noted that VR-based RT provided a shared platform for communication, making it easier for caregivers and people with dementia to connect over mutual experiences.

### VRRT and Its Impact on Cognitive Stimulation

While RT via VR has shown promising effects on emotional well-being and social engagement, its impact on cognitive function remains inconclusive. In particular, a study suggests that RT via VR may help preserve cognitive function during the intervention period, but its long-term effects are unclear, requiring further research to determine whether these benefits persist over time [32].

On the other hand, several studies found no significant improvements in cognitive domains such as attention, memory retention, or processing speed, nor sufficient evidence that VR-based RT reduces cognitive decline over time [27,35,37]. However, people with dementia with higher levels of apathy exhibited significant cognitive improvements following RT via VR, suggesting that the therapy may be particularly beneficial for specific subgroups [38,39].

One possible explanation for these findings is that RT via VR primarily stimulates emotionally charged autobiographical memories, rather than engaging higher-order cognitive processes such as reasoning, problem-solving, or working memory [35,39]. While RT is known to activate episodic memory networks, the extent to which these activations contribute to broader cognitive function remains uncertain [27,39].

Additionally, RT via VR was found to elicit autobiographical memories in familiar environments, reinforcing the importance of personalized content [30,33]. This suggests that the success of RT via VR may depend on familiarity with the virtual environment, with well-known settings enhancing reminiscence and memory recall more successfully than unfamiliar ones.

### Current Limitations and Future Directions in VRRT

Despite the promising potential of using VR to enhance RT, several limitations hinder its widespread implementation in dementia care settings. One of the primary challenges identified in the findings is VR technology’s high cost and resource-intensive nature. Many long-term care facilities lack the financial resources to invest in expensive VR headsets, high-quality software, and the necessary infrastructure for setup and maintenance [40]. While SI-VR solutions using large screens provide a more affordable alternative, they lack the same level of immersion and engagement as FI-VR [35,36]. Additionally, RT via VR requires dedicated space and structured session planning, which can be difficult for care facilities with limited resources and understaffed teams. To address these issues, research should focus on developing cost-effective VR solutions for RT, such as mobile-based VR apps and lightweight, affordable VR headsets that require minimal setup [36]. Additionally, open-source VR software and cloud-based VR platforms could reduce infrastructure costs and improve accessibility for lower-resource care settings [40]. Scalable VR solutions with portable, low-cost hardware and preprogrammed virtual environments could make VR more practical for widespread clinical use [37].

Another significant limitation is the technical complexity of VR systems and the need for caregiver training. Facilitating an RT session using VR systems involves specialized equipment that requires caregivers to be trained in setup, troubleshooting, and guiding people with dementia through VR experiences [27,40]. However, many caregivers report limited confidence in using technology, and the high turnover rates in dementia care settings make continuous training difficult [37,40]. To improve usability and adoption, VR developers should focus on creating simplified, user-friendly interfaces that caregivers can operate with minimal training [16]. Structured VR training modules for caregivers, including interactive tutorials and hands-on workshops, could further support the implementation of VR in dementia care [31]. Future research should explore the integration of voice-assisted navigation and automated session setup, allowing caregivers to facilitate VR for RT with minimal assistance [27].

The findings also indicate that people with dementia exhibit individual variability in their response to VR, with some experiencing sensory overload, disorientation, or fatigue, making it necessary to adjust VR exposure based on individual needs [27,28,31]. However, many VR systems in dementia care lack adaptive features that personalize the therapeutic content based on cognitive and sensory preferences. Thus, adaptive VR experiences that adjust based on people with dementia’s emotional and cognitive responses could help tailor RT sessions via VR to individual needs, reducing the risk of sensory overload [33].

Additionally, there is limited research on the long-term effects of VR in RT and its ability to sustain therapeutic benefits over time. While multiple studies confirm that VR can improve RT’s outcomes, there is insufficient evidence on whether it provides lasting improvements [27]. Most existing studies focus on short-term effects, with limited follow-up on how repeated exposure to VR influences neurocognitive resilience in people with dementia. Furthermore, the effectiveness of VRRT compared to traditional RT remains unclear, raising questions about its long-term clinical value and cost-effectiveness [40]. Future research should prioritize longitudinal studies that assess the sustained effects of VR in RT over time [35]. Lastly, comparative studies evaluating VRRT against traditional RT could provide deeper insights into its long-term therapeutic potential and clinical relevance [36,40].

## Discussion

### Principal Findings

The findings of this review suggest that RT via VR is feasible and can positively enhance intervention in dementia care, with notable benefits in emotional well-being and social engagement. In particular, and in response to RQ1, it was found to be a feasible and well-tolerated intervention for people with dementia. Studies consistently report that people with dementia can successfully engage with virtual environments without experiencing major adverse effects. The availability of both fully immersive and semi-immersive systems enhances its adaptability, making it accessible for people with dementia with mild, moderate, and severe degrees of cognitive impairment and mobility limitations.

RT via VR has also been shown to enhance the health-related quality of life for people with dementia. In response to RQ2, the findings indicate that RT via VR addresses the BPSD by reducing agitation, anxiety, and apathy, while fostering relaxation and positive emotions, especially when immersed in peaceful, familiar, and personally meaningful VR environments. It is worth mentioning that the impact of RT via VR on cognition remains inconclusive. Studies examining its effects on memory recall, attention, and executive function found no significant improvements in cognitive performance. While RT via VR is able to stimulate autobiographical memory retrieval, it does not necessarily enhance long-term cognitive function or slow disease progression.

### Comparison to Prior Work

The findings of this review are broadly consistent with recent reviews, both systematic and scoping, evaluating VRRT among older adults, including those with cognitive impairment. A systematic review [43] reported that VRRT is associated with emotional benefits such as reductions in anxiety, apathy, and depressive symptoms. Similarly, scoping reviews [26,27] found that immersive, autobiographically meaningful content tends to enhance mood and emotional engagement. However, this review also makes several distinct contributions to the literature. First, it focuses exclusively on people with dementia, while prior reviews included broader populations, such as older adults with or without cognitive impairment [26,27]. This more specific scope offers a dementia-targeted synthesis, enabling better applicability for practitioners and researchers in dementia care. Second, this review provides a more context-sensitive analysis of implementation challenges, extending beyond general usability concerns, identified in previous reviews such as simulator sickness, interface complexity, and the need for caregiver assistance [26,43]. This review builds upon prior usability discussions by identifying additional practical barriers that influence VRRT implementation, including the need for trained personnel, space constraints, and technical support requirements. While earlier reviews report general challenges, this review highlights how these factors can vary across care settings, suggesting the importance of context-specific implementation planning.

The cognitive outcomes reported remain limited across all reviews. While some evidence suggests that VRRT may stimulate autobiographical memory in the short term, none of the included reviews, including this one, found robust support for long-term improvements in memory, attention, or executive functioning. In previous reviews, the absence of randomized controlled trials to evaluate the solution has been emphasized [26,43], along with the need for more rigorous data [27]. Building on these insights, this review advocates for designing future studies around unified cognitive metrics and sustained postintervention follow-ups.

Lastly, this work offers forward-looking insights by providing practical, design-oriented recommendations, such as integrating mobile VR platforms, simplifying user interfaces, and creating culturally adaptive content libraries. These suggestions are aimed at addressing cost, accessibility, and caregiver usability gaps, areas not thoroughly operationalized in previous reviews [26,27]. As such, this review serves not only as a synthesis of existing evidence but also as a strategic roadmap for designing more scalable and feasible VRRT interventions explicitly tailored for dementia care.

### Strengths and Limitations

This review provides a comprehensive synthesis of current evidence on the feasibility, acceptability, and therapeutic potential of delivering RT through VR for people with dementia. By incorporating studies that span varying levels of cognitive impairment and care environments, it offers a nuanced understanding of how both SI-VR and FI-VR technologies can be adapted to meet the complex and evolving needs of this population. A key contribution of this review lies in its focused analysis of critical dimensions often overlooked in prior reviews, including the degree of immersion, personalization, content relevance, and implementation challenges within real-world care settings.

To the best of our knowledge, this is the only recent review that concentrates solely on people with dementia, excluding broader populations of older adults with or without cognitive impairment, while examining the differential use of SI-VR and FI-VR systems. Moreover, by emphasizing system design considerations, the review aligns with contemporary calls for more inclusive, accessible, and person-centered VR interventions. These insights extend the review’s relevance beyond clinical practitioners to include gerontologists, human-computer interaction researchers, and technology developers working at the intersection of dementia care and digital innovation. In doing so, it contributes a timely and interdisciplinary perspective that can guide the design, deployment, and evaluation of future VR-based therapeutic tools.

However, in response to RQ3, several limitations must be acknowledged. First, despite the promising findings, the high cost of VR equipment, software, and required infrastructure remains a significant barrier to widespread adoption, especially in resource-limited care settings. While semi-immersive systems may offer more accessible alternatives, they often lack the engagement and immersive quality necessary to maximize therapeutic effects. Second, the technical complexity of VR systems presents an operational challenge. Caregivers must receive adequate training to operate equipment and support people with dementia through virtual experiences, which may be unrealistic in understaffed environments.

Furthermore, many of the included studies are limited by small sample sizes, short intervention durations, and a lack of standardized outcome measures, which hinders cross-study comparability. Research on the long-term efficacy of VRRT is particularly scarce, with most studies focusing on immediate or short-term outcomes. The variability in individual responses also complicates implementation; while some people with dementia benefit substantially, others may experience sensory overload, fatigue, or disengagement. Current VR platforms generally lack adaptive features that can tailor the experience to individual user profiles or adjust dynamically in response to behavioral feedback.

In addition to the challenges identified in response to RQ3, this review itself is subject to several methodological limitations. First, the literature search was limited to freely accessible full-text papers published in English. As a result, relevant studies published behind paywalls or in other languages may have been excluded, potentially narrowing the comprehensiveness and representativeness of the findings. Second, the review was conducted by a single author, who carried out all stages of the process, including study screening, selection, and data extraction. While efforts were made to apply clear and consistent criteria throughout, the absence of a second reviewer may have introduced potential selection bias and reduced intercoder reliability.

### Future Directions

To address RQ4, future research should enhance affordability, personalization, long-term impact, and usability of VRRT for people with dementia. Developing cost-effective, mobile-based VR solutions, such as lightweight headsets or tablet-compatible apps, could significantly enhance accessibility, particularly in underresourced care settings. These alternatives should aim to retain therapeutic immersion while reducing financial and infrastructural burdens associated with high-end VR systems.

Moreover, the personalization of VRRT experiences remains critical to their effectiveness. Future systems should integrate adaptive features that tailor content based on individual life histories, preferences, and cognitive or sensory needs. Approaches such as user-driven content selection, biometric feedback integration, and modular content frameworks can help dynamically adjust the experience to match user tolerance and emotional state.

To maximize real-world applicability, simplified interfaces and structured caregiver training protocols are necessary to reduce technical complexity and empower care staff. The inclusion of training toolkits, step-by-step onboarding modules, and in-app guidance systems could support smoother integration in everyday care workflows.

Importantly, research should also focus on establishing the long-term effects of VRRT through high-quality, longitudinal studies. These should investigate not only sustained psychological and cognitive outcomes but also the potential for reducing caregiver burden and improving overall health-related quality of life. Comparative trials between VRRT and traditional RT are also needed to clarify cost-effectiveness and relative efficacy.

Finally, co-design methodologies that involve people with dementia, caregivers, and interdisciplinary experts in the development process can ensure that future VRRT systems are ethically grounded, emotionally safe, and attuned to the lived realities of diverse dementia care populations.

### Conclusions

In conclusion, this scoping review illustrates that the employment of RT through VR provides a viable and innovative strategy for dementia care. Nevertheless, practical challenges such as considerable costs, the complexity of personalization, and the necessity for caregiver training impede its extensive implementation within care environments. Notwithstanding these obstacles, the findings highlight the potential of VRRT to function as a scalable, person-centered tool in dementia care. Subsequent research that addresses usability, cost-effectiveness, and long-term outcomes, while integrating inclusive co-design methodologies, can contribute to the evolution of VRRT into a sustainable and equitable element in dementia care.

## Supplementary material

10.2196/73539 Checklist 1 PRISMA-ScR. PRISMA-ScR: Preferred Reporting Items for Systematic Reviews and Meta-Analyses Extension for Scoping Reviews.

## Abbreviations

BPSD: behavioral and psychological symptoms of dementia
FI-VR: fully immersive virtual reality
HMD: head-mounted display
PRISMA-ScR: Preferred Reporting Items for Systematic Reviews and Meta-Analyses Extension for Scoping Reviews
RQ: research question
RT: reminiscence therapy
SI-VR: semi-immersive virtual reality
VR: virtual reality
VRRT: virtual reality reminiscence therapy
